# Experimental validation of a subject-specific finite element model of lumbar spine segment using digital image correlation

**DOI:** 10.1371/journal.pone.0272529

**Published:** 2022-09-09

**Authors:** Chiara Garavelli, Cristina Curreli, Marco Palanca, Alessandra Aldieri, Luca Cristofolini, Marco Viceconti

**Affiliations:** 1 Department of Industrial Engineering, Alma Mater Studiorum—University of Bologna, Bologna, Italy; 2 Medical Technology Lab, IRCCS Istituto Ortopedico Rizzoli, Bologna, Italy; Medical College of Wisconsin, UNITED STATES

## Abstract

Pathologies such as cancer metastasis and osteoporosis strongly affect the mechanical properties of the vertebral bone and increase the risk of fragility fractures. The prediction of the fracture risk with a patient-specific model, directly generated from the diagnostic images of the patient, could help the clinician in the choice of the correct therapy to follow. But before such models can be used to support any clinical decision, their credibility must be demonstrated through verification, validation, and uncertainty quantification. In this study we describe a procedure for the generation of such patient-specific finite element models and present a first validation of the kinematics of the spine segment. Quantitative computed tomography images of a cadaveric lumbar spine segment presenting vertebral metastatic lesions were used to generate the model. The applied boundary conditions replicated a specific experimental test where the spine segment was loaded in compression-flexion. Model predictions in terms of vertebral surface displacements were compared against the full-field experimental displacements measured with Digital Image Correlation. A good agreement was obtained from the local comparison between experimental data and simulation results (R^2^ > 0.9 and RMSE% <8%). In conclusion, this work demonstrates the possibility to apply the developed modelling pipeline to predict the displacement field of human spine segment under physiological loading conditions, which is a first fundamental step in the credibility assessment of these clinical decision-support technology.

## Introduction

Vertebral fracture is one of the most serious orthopaedic injuries, associated with several adverse consequences including back pain, disability, risk of neurological complications, increased risk of death and a decreased quality of life [1, 2]. These fractures are typically traumatic or pathological (the latter being sometime referred in the medical literature with fragility fractures), or more often a combination of both factors. Conditions like osteoporosis, or the presence of metastatic lesions weaken the vertebral body and increase the risk of fragility fractures [2–4]. The incidence of vertebral fragility fractures in women over 50 is around 1%, [5] and it is expected to increase in the next years because of the aging population. Moreover, epidemiology studies show that only about one-third are clinically diagnosed [6, 7]. Physical therapies, pharmacological treatments, and minimally invasive vertebral augmentation techniques such as kyphoplasty and vertebroplasty are usually used to improve the biomechanical strength of the pathological vertebral body. However, because of several possible clinical complications, the choice of treatment is complex and providing a reliable estimate of the risk of fracture is fundamental to support the clinical decision.

Several tools and indexes were proposed in the last decades to predict the risk of vertebral fracture [2]. However, the gold standard in the clinical practice, the Vertebral Fracture Assessment (VFA), is a diagnostic method to detect fractures that already occurred and can be used as an indicator for subsequent fractures only. Dual-energy X-ray absorptiometry (DXA) can measure bone mineral density (BMD) that indeed correlates with bone strength [8], even if it does not directly measure it. However, DXA-derived BMD measurement can explain only around the 60% of the variation in vertebral strength [9], because it does not take into account other elements that concur in bone strength assessment, such as inhomogeneity of density distribution and complexity of bone geometry. A promising tool to evaluate vertebral fracture risk is Biomechanical Computed Tomography (BCT) [10], widely and successfully used to provide a measurement of bone strength at the hip. *Subject-specific* finite element (FE) models are created based on quantitative computed tomography (QCT) images and used to compute the force required to virtually fracture the vertebral body [11–16]. The models demonstrated to be more accurate in predicting vertebral strength and stiffness compared with dual-energy x-ray absorptiometry [17]. The BCT works under the assumption that the disease affecting the skeleton modified the quantity of bone tissue, but not its quality. This is true for diseases like osteoporosis or metastatic disease of lytic type, but could show limitations for other diseases such as osteogenesis imperfecta or blastic metastases. Therefore, the BCT workflow can also be applied to metastatic bone where the lytic nature of the disease is prevalent.

One of the most crucial aspects that must be properly addressed before applying the BCT technology in the clinical practice is the model validation [18]. Validation aims at determining the predictive capability of a computational model for its intended use and is usually done by comparing the predicted results against experimental data [19]. Several studies focused on this specific problem and different validation metrics were used. Comparisons between computational results and experimental measurements have been performed comparing global mechanical properties [11, 13, 14, 17]. Imai et al., for example, considered four mechanical properties to evaluate the accuracy of the developed FE model: yield load (*r* = 0.949), fracture strength (*r* = 0.978), fracture site and minimum principal strain on the surface of the vertebra (*r* = 0.838) [11]. In the comparative study presented by Buckley at al. [14], good agreement was found for vertebral strength (*R*^2^ = 0.80) while axial stiffness was predicted less well (*R*^2^ = 0.27). Also Dall’Ara et al. in their validation study [13], reported stronger correlation for strength (*R*^2^ = 0.79) compared to stiffness (*R*^2^ = 0.49). Only recently, more detailed and specimen specific validation metrics based on the comparison between predicted and measured displacements extracted on the vertebral bodies with Digital Volume Correlation (DVC) and Digital Image Correlation (DIC) were presented [15, 20]. In the study published by Jackman et al., the accuracy of QCT-based FE analyses in predicting vertebral failure patterns was evaluated on three-vertebrae thoracic spine segments comparing the predicted displacements with those measured by DVC. They studied different boundary conditions, loading modes and yield criteria capturing some of the qualitative features of the failure patterns (e.g., vertebral deformation during flexion loading). Gustafson et al. used DIC technique for measuring the displacement fields, and found good FE-experimental agreement (R^2^ = 0.75–0.93 for the surface displacements field and R^2^ = 0.90 for the specimen stiffness), demonstrating the possibility to use a non-contact optical full-field measurement technique for the validation of vertebral FE models, as also already confirmed for human femur applications [21–23]. However, a critically important aspect must be considered: in [15], compression tests were performed on a single vertebra without intervertebral discs; this choice altered the loading and boundary conditions that may create artifacts (e.g., non-physiological failure mechanics) and neglects what happen *in vivo*. A more clinically relevant validation can be obtained considering at least two spinal units segment so that the load is more physiologically transferred to the vertebral body through the intervertebral discs, as also reported in a recent work [16].

In authors’ knowledge, there are no studies in the literature that describe in detail a reliable procedure to validate *subject-specific* FE models of the spine segment using experimental measurements obtained with DIC. Among the possible reasons there is the difficulty in developing complex three-dimensional FE models of the spine and in accurately reproducing physiological loading conditions defined in the experimental tests. Also, while there is extensive evidence that the attenuation coefficients that a CT provides are correlated to the elastic modulus of the mineralised tissue, there are currently no ways to estimate the biomechanical properties of intervertebral disc from CT images. Thus, an idealised constitutive equation to model the disc should be considered.

The aim of this paper is to present the validation of a multi-vertebrae spine model based on the comparison between the vertebral full-field surface displacements predicted by the FE model against the experimentally measured ones using DIC. In case of multi-vertebrae specimen, it is important to verify that the model reproduces the correct spine kinematics. Considering that the prediction of bone deformation is influenced by the relative movement between two adjacent vertebrae and the overall spine motion, this work is a fundamental preliminary step towards the full validation of spine models.

A cadaveric lumbar spine segment presenting osteoporosis in one vertebral segment, and a metastatic lesion in another was used as case study to demonstrate the possibility to apply the procedure to clinically relevant scenarios.

## Materials and methods

### Experimental procedure

**Fig 1:**
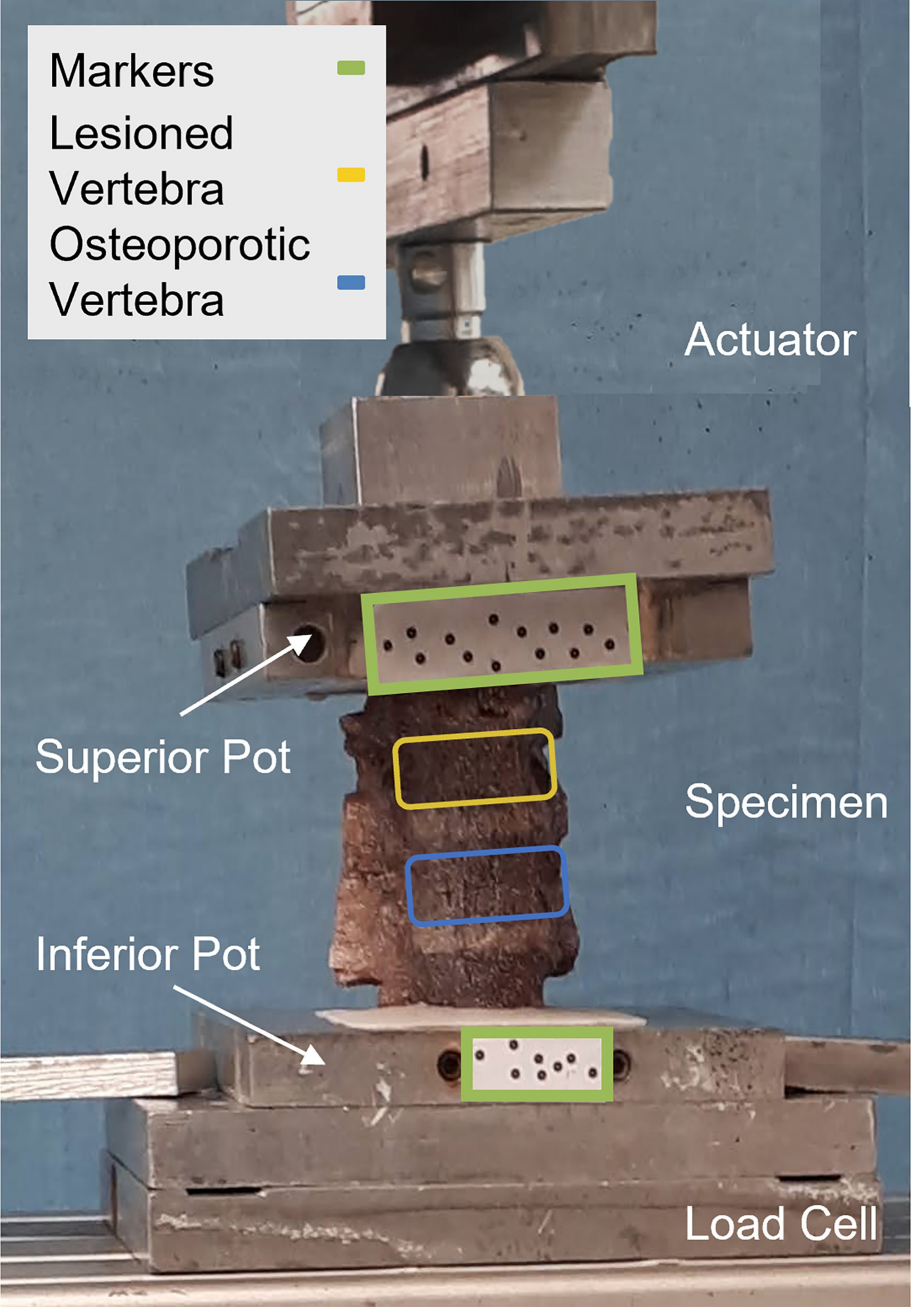
Experimental set up. Specimen mounted on the testing machine with the set up in the compression-flexion configuration.

The study has been approved by the ethical committee of the University of Bologna (n. 17325, 08/02/2019). The test was performed in accordance with the Declaration of Helsinki on a cadaveric spine obtained from an ethically approved donation program (Anatomic Gifts Registry, USA), which requires legally valid informed consent. The subject was a 73-year-old female with a body weight of 72.6 kg, affected by lung cancer with spine metastasis. Lumbar segment from L1 to L4 was extracted from the spine: L2 was evaluated by two expert clinicians as metastatic [24] while L3 did not show any radiographical signs of metastasis. The top half of the most cranial vertebra (L1) and the bottom half of the most caudal vertebra (L4) were embedded in polymethyl-methacrylate (PMMA) (Fig 1) to be mounted on a uniaxial testing machine. The entire segment was aligned following the procedure described by Danesi et al. [25].

Tomograms of a European Spine Phantom (ESP) and of the specimen were acquired with a CT (AquilionOne, Toshiba, Japan) with the following parameters: tube current 200 mA, voltage 120 kVp, without any current compensation. The voxel dimension was 0.24 x 0.24 x 1 mm. The densitometric analysis of the calibrated CT images showed few regions in the L2 segment with mean volumetric Bone Mineral Density (vBMD) of 105 mg/cm^3^, about 5 times less than the vBMD measured in the whole vertebral body. On the other hand, the L3 vertebral segment, with its average vBMD of 76 mg/cm^3^, would be classified as osteoporotic according to the ACR classification [26]. The two vertebrae will be referred hereinafter as lesioned (L2) and osteoporotic (L3).

The experimental protocol used to test the specimen was reported in detail by Palanca et al. [4]. Briefly, the mechanical test was performed using a uniaxial testing machine (Instron 8500 controller with Instron 25 kN load cell, Instron, UK). A random speckle pattern was prepared on the specimen and anoptimized three-dimensional Digital Image Correlation 4-camera system (Aramis Adjustable 12M, GOM, Braunschweig, Germany, with 12MPixels cameras and 75 mm metrology-standard lenses), was used to measure the displacement and strain fields.

In order to load the specimen in compression-flexion, the application point of the force (*F*) was offset toward anterior by 4 mm, i.e., 10% of the antero-posterior dimension (*d*) of the L2-L3 intervertebral disc, similar to [27]. To perform the compression-flexion, avoiding any transmission of undesired load components, the superior pot was free to rotate and translate by means of a ball-joint and two low-friction orthogonal linear bearing, while the inferior pot was fixed [28]. The target load to be applied was tuned to reach an average compressive strain of -2500/-3500 microstrain on the anterior surface of L3 vertebral body (monitored in real-time through the DIC system). This is the strain range associated to physiological load [29]. The specimen was tested applying a monotonic ramp that reached the target load (60 N) in 1.0 second. According to the testing machine report, the uncertainty on the applied load was around 4 N (0.14% at 2500N). Moreover, the auto-range of the testing machine control allows to reduce it. During the test, images of the spine segment were recorded at 25 Hz to measure the full-field displacement and strain on the external surface of L2 and L3 vertebral bodies, following the procedure described in [30]. Processing of the images was performed using a facet size of 30 pixels, a grid spacing of 10 pixels, a median spatial filter on 5 facets, and a median temporal filter on 2 frames. These parameters allowed us to obtain a measurement spatial resolution of about 2 mm, with a strain systematic error of 30 microstrain and a strain random error of 100 microstrain. Systematic and random errors in term of displacements were found around 10 μm and 25 μm, respectively. Flat circular markers were glued on the aluminium pots of the test machine to track the displacement of the superior and inferior pots (Fig 1).

### Computational procedure

Finite element simulations were performed considering subject-specific models of the vertebral bodies and population-averaged models of the intervertebral discs (IVDs).

To create the geometry, all the specimen components (two entire vertebral bodies, two half vertebral bodies and three IVDs) were firstly segmented individually from the CT-images using a threshold-based algorithm with lower and upper Hounsfield Unit (HU) values set to 226 and 3071 respectively, and manual editing (Mimics, Materialise NV, Leuven, Belgium). Solid parts were reconstructed, and body attachments were created through Boolean operations (SpaceClaim V19.3, Ansys Inc., Canonsburg, PA). A mesh of 10-node iso-parametric quadratic tetrahedral elements was generated using an Octree automatic mesh generation algorithm (ICEM CFD V19.3, Ansys Inc.). Octree meshing was selected because it is more robust to topological imprecisions, and thus can be used to mesh directly the polygonal surfaces obtained from the CT-images segmentation. The mesh was generated by imposing a max element edge length equal to 2 mm. The mesh size was selected based on a preliminary convergence test, where the maximum displacement in the two regions of interest change of only 0.01% with a further refinement of the mesh.

Bone tissue was modelled as an heterogenous, locally isotropic, linear elastic brittle material. While more complex formulations have been proposed, this constitutive equation allowed the prediction of bone strains with an accuracy of 93% [31]. The heterogenous elastic properties were derived from the CT data, assuming them related to the density of the mineral phase in each point of the bone. The elastic properties of the bone were subsequently mapped on each element (Bonemat® V3.1, Istituto Ortopedico Rizzoli, Bologna, Italy) [32] after converting the HU values of CT images voxel into volumetric bone mineral density equivalent values (ρ_QCT_) using the following calibration equation, specific for the ESP previously scanned:

$$\rho_{QCT}=-0.016404+0.00085164\cdot HU$$ (1)

The density to elasticity relationships (Eqs 2 and 3) were adopted to convert ρ_QCT_ to ash density (ρ_ash_) as proposed by Schileo et al. [31] and then to the elastic modulus (*E*) following the equation provided by Morgan [33], with ρ_app_ = ρ_ash_/0.6 according to [31]. Poisson’s ratio was set to 0.3. In the Eqs 2 and 3 E is expressed in MPa and ρ in g/cm^3^.


$$\rho_{ash}=0.079+0.877\cdot \rho_{QCT}$$ (2)

$$E=4730\cdot \rho_{app}{}^{1.56}$$ (3)

Both osteoporotic and lesioned vertebrae were modeled in the same way, according to [34, 35]. The intervertebral discs were modelled as a single homogeneous isotropic material with a Poisson’s ratio equal to 0.1, as proposed in [36]. The modulus of elasticity was calibrated for the specified loading condition so that the global stiffness of the spine segment, as predicted by the finite element model, matched that measured experimentally. A percentage difference less than 0.1% computed between the predicted axial load and the force measured experimentally was considered acceptable for the study. The resulting modulus of elasticity was 1.92 MPa, which is within the range of values reported in the literature [36].

**Fig 2:**
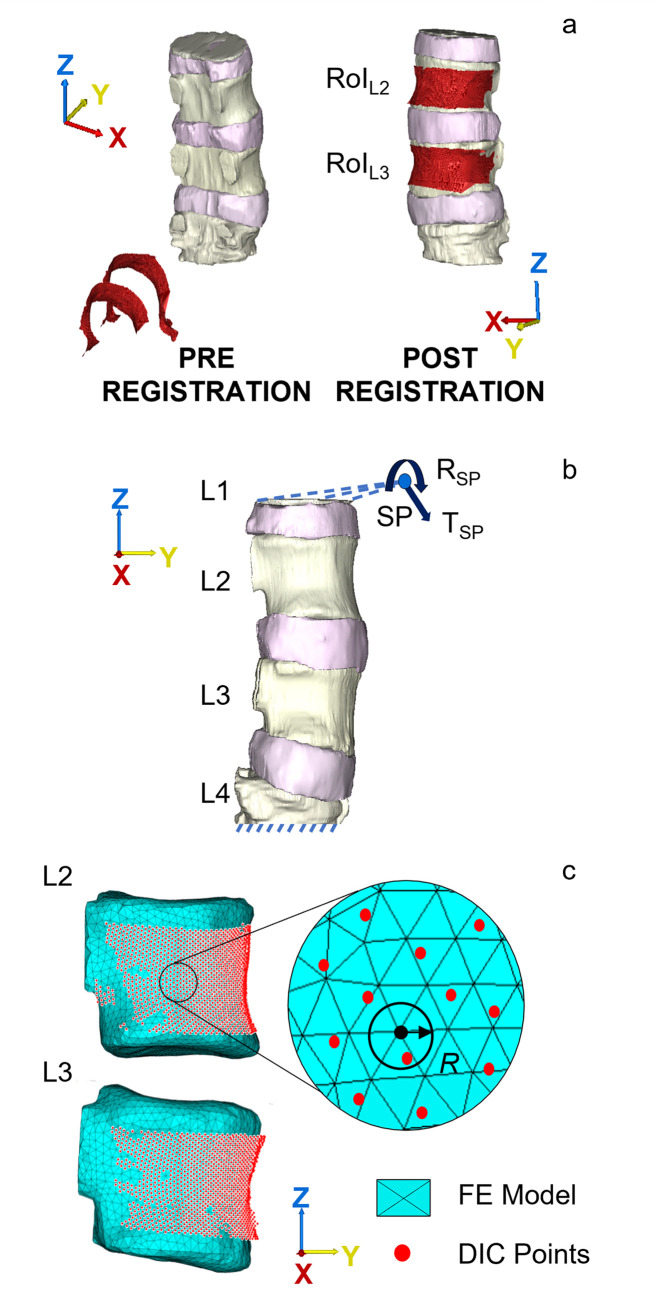
Workflow used to compare predicted and experimental local displacements. a) Registration of the DIC points (red surfaces) to the segmentation obtained from the CT data. By aligning the surfaces, it was possible to perform the comparison between the displacements predicted by the FE model and the ones measured using the DIC. b) Boundary conditions applied to the FE model: the inferior surface of the lower vertebral body was fixed while translations (T_SP_) and rotations (R_SP_) are applied through a pilot node SP to the superior surface of the upper vertebral body. c) Illustration of the tool used for the local displacement comparison.

After the generation of the model, a rigid registration was performed using a feature-based rigid registration algorithm (Mimics, Materialise NV) to align the experimental DIC data of the two free vertebral surfaces at the initial (unloaded) configuration to the segmentation obtained from the CT data (Fig 2A). Since the registration tool required a polygonal surface as mover body, a Delaunay triangulation algorithm (Matlab® v2020, MathWorks, Natick, Massachusetts, US) was previously used to transform the DIC point cloud into an open triangulated surface. The rigid transformation matrix (Mt) was then extracted using a single value decomposition algorithm (Matlab® v2020, MathWorks, Natick, Massachusetts, US) that best align the original and the registered DIC point clouds. All the displacement vector components of all the correlated DIC points and the coordinates of the markers placed on the superior and inferior pots were transformed using Mt.

The registration accuracy was assessed computing the Euclidean distance between the points on the DIC-detected surface and the nearest corresponding node on the model surface (Registration Error) and was expressed as the root mean square error (RMSE).

The boundary conditions (BCs) simulated the experimental test (Fig 2B). Particularly, the inferior surface of the lower vertebral body (L4) was fixed while the rigid body motion of the superior aluminium pot, extracted using the single value decomposition algorithm [37], was transferred at the superior surface of the upper vertebral body (L1) using a remote displacement approach (Mechanical APDL V19.3, Ansys Inc.). Both displacements and rotations were prescribed through a pilot node (SP) that corresponds to the centroid of the registration marker cluster placed on the metal pot (Fig 1). The remote boundary conditions were implemented with the Multipoint Constraint (MPC) technique using the rigid surface-based constraint. The computed translation vector ***T***_***SP***_ (expressed in mm) and the rotation matrix R_SP_ are reported below:


$$T_{SP}=\left[\begin{array}{c} 1.321\\ 5.441\\ -4.754 \end{array}\right]\;R_{SP}=\left[\begin{array}{ccc} 1 & 0.014 & 0.005\\ -0.014 & 0.996 & 0.088\\ -0.003 & -0.088 & 0.996 \end{array}\right]$$ (4)

To investigate the model results and allow the comparison with the experimental data, two regions of interest RoI_L2_ and RoI_L3_ (respectively red and green contours in Fig 2B) were defined on the anterior and lateral surface of the two vertebral bodies L2 and L3. These regions correspond to the correlation areas where displacements are captured by the DIC system during loading. In addition to the three displacement components, the axial resultant force was computed as a constraint node reaction solution at bottom surface of the lower vertebra.

The entire FE model had 1,198,125 degrees of freedom and took approximately 2 minutes to solve with Ansys preconditioned conjugate gradient solver, using parallel distributed memory over 6 cores with 64 GB of RAM (Intel(R) Xeon(R) E-2276G CPU 3.80GHz).

### Statistics

To compare the simulation results with the experimental data, the DIC displacement measurements were first averaged over a spherical volume with radius R equal to the registration RMSE (see result and discussion section). Then, prediction accuracy was evaluated comparing the displacements predicted by the FE model at each node of the RoI (**U**_**FEM**_) against the measured displacement averaged over the spherical volume of interest centered on that node (**U**_**DIC**_) (Fig 2C). The total number of experimental data points used for the validation was 2,452 (1,143 and 1,309 for L2 and L3 vertebral body respectively). Cook’s distance method was used to clean data from potential outliers. Specifically, points with a Cook’s distance greater than four times the mean Cook’s distance value were removed from the comparison [38]. Linear regressions were used to estimate the goodness of the comparisons: determination coefficient (R^2^) was used to quantify how well a linear model described the FE to DIC relationship, while RMSE was chosen as a good measure of how accurately the model predicts the response. A normalized RMSE (%RMSE) was calculated using the maximum displacement measured by DIC in each RoI as normalization factor. The absolute error and the percentage error were computed at each point and for every directional component as follow:

$${Error}_{x,y,z}=|U_{{DIC}_{x,y,z}}-U_{{FEM}_{x,y,z}}|$$ (5)

$${Error}_{x,y,z}\%=\frac{\left|U_{{DIC}_{x,y,z}}-U_{{FEM}_{x,y,z}}\right|}{\left|U_{{DIC}_{x,y,z}}\right|}\cdot 100$$ (6)

Maximum values of the absolute error (Max Error) and average values of the percentage error (Average Error %) were then extracted in each RoI. The vector resultant (Diff) was also computed as:

$$Diff=\sqrt{Erro{r_{x}}^{2}+Erro{r_{y}}^{2}+Erro{r_{z}}^{2}}$$ (7)

## Results

**Fig 3:**
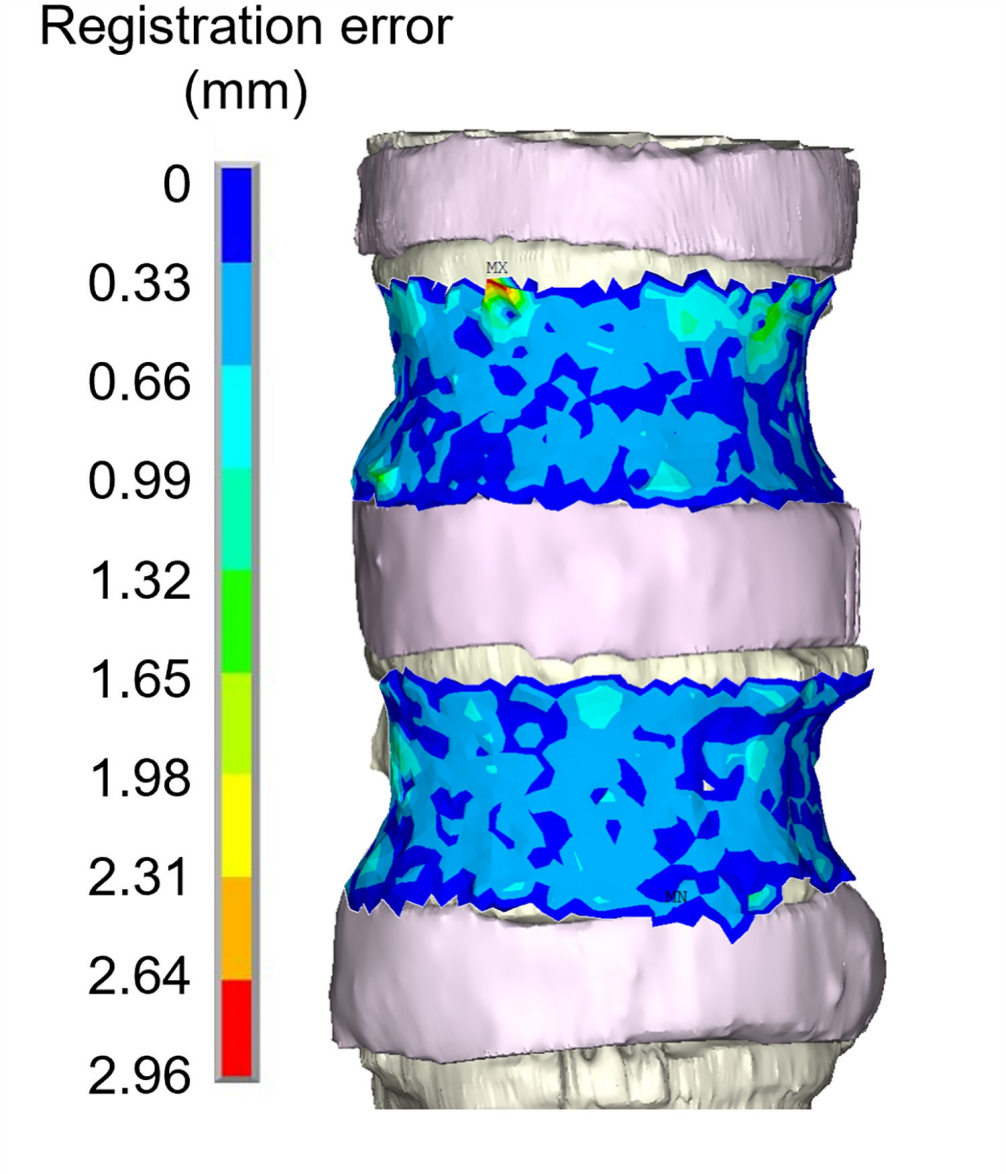
Graphical representation of spatial distribution of the registration error. Spatial distribution of the registration error of DIC points on the vertebral surfaces of the FE model.

After registration, DIC surface points and FE external nodes, both extracted in the unloaded condition, had a least square distance of 0.34 mm and a registration RMSE equal to 0.53 mm. A maximum distance of 2.96 mm was found between the DIC points and the corresponding nodes of the model near the upper endplate of the L2 vertebra (Fig 3).

**Fig 4:**
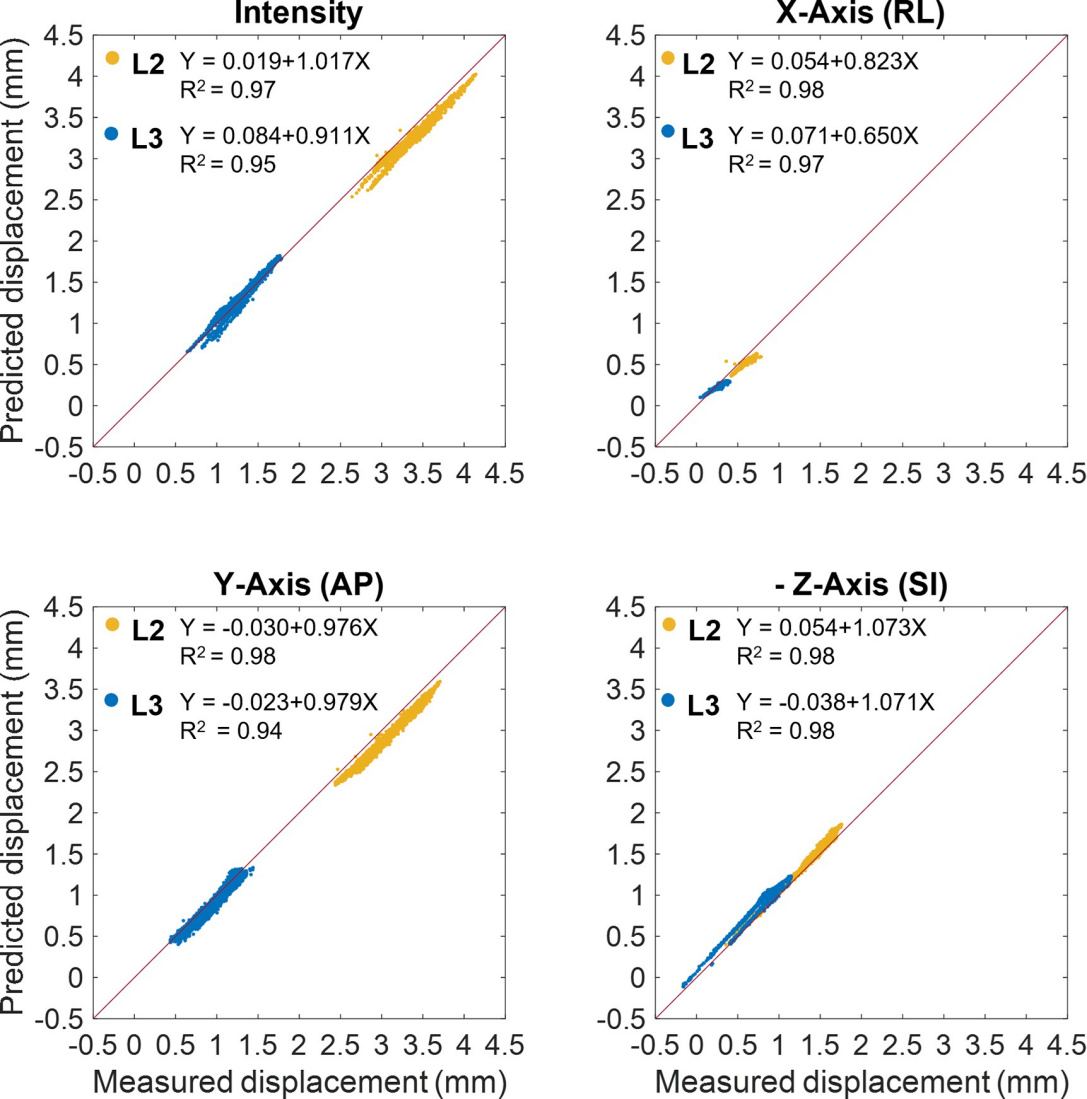
Linear regressions plots between predicted and experimental local displacements. Comparison between the displacements measured using DIC and the ones predicted by the FE model for L2 (yellow) and L3 (blue) vertebral surfaces. The Euclidean norms of the displacements, referred as Intensity, and the three displacement components defined in the FE model coordinate reference system are plotted. Linear regression and coefficient of determination are also reported for the correlation plots. It is important to notice that the SI component is plotted with the sign changed to allow comparison with the other subplots.

**Fig 5:**
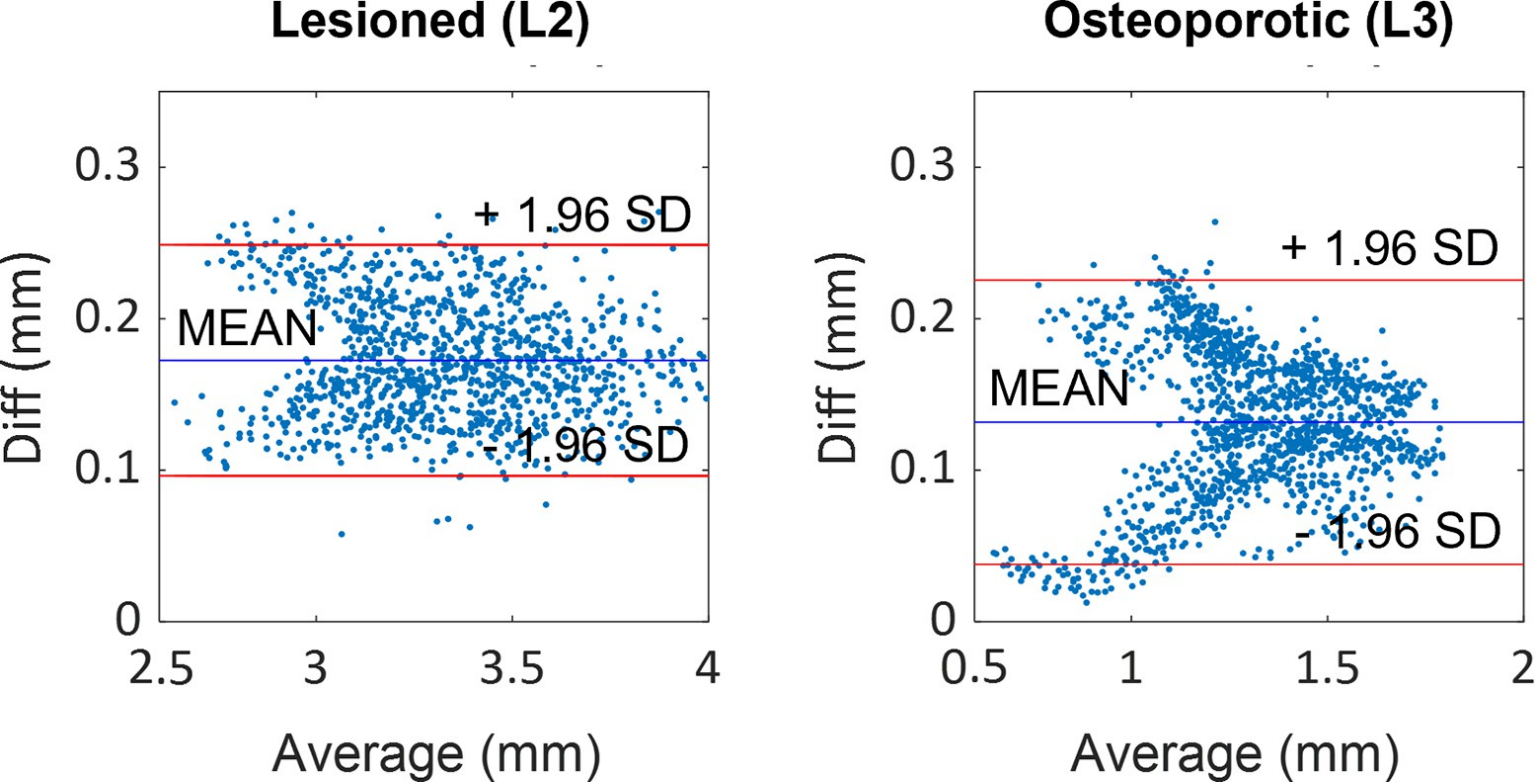
Residual analysis estimated between FE and DIC displacements. Bland-Altman plots showing the dependence of the vector sum magnitude (Diff) on the average value between measured and predicted local displacement.

**Fig 6:**
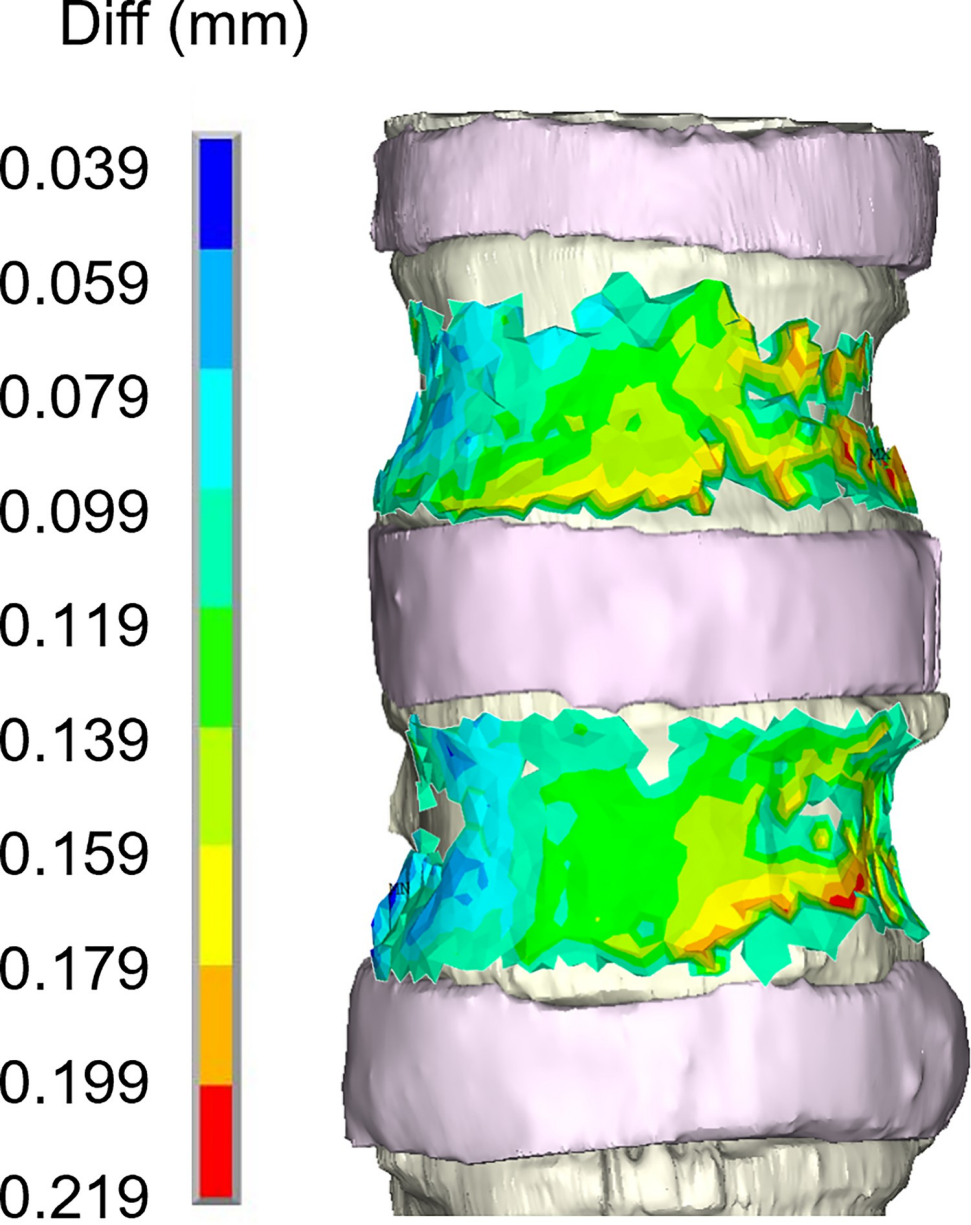
Representation of comparison error spatial distribution. Spatial distribution of the vector resultant (Diff) on the vertebral surfaces of the FE model.

**Fig 7:**
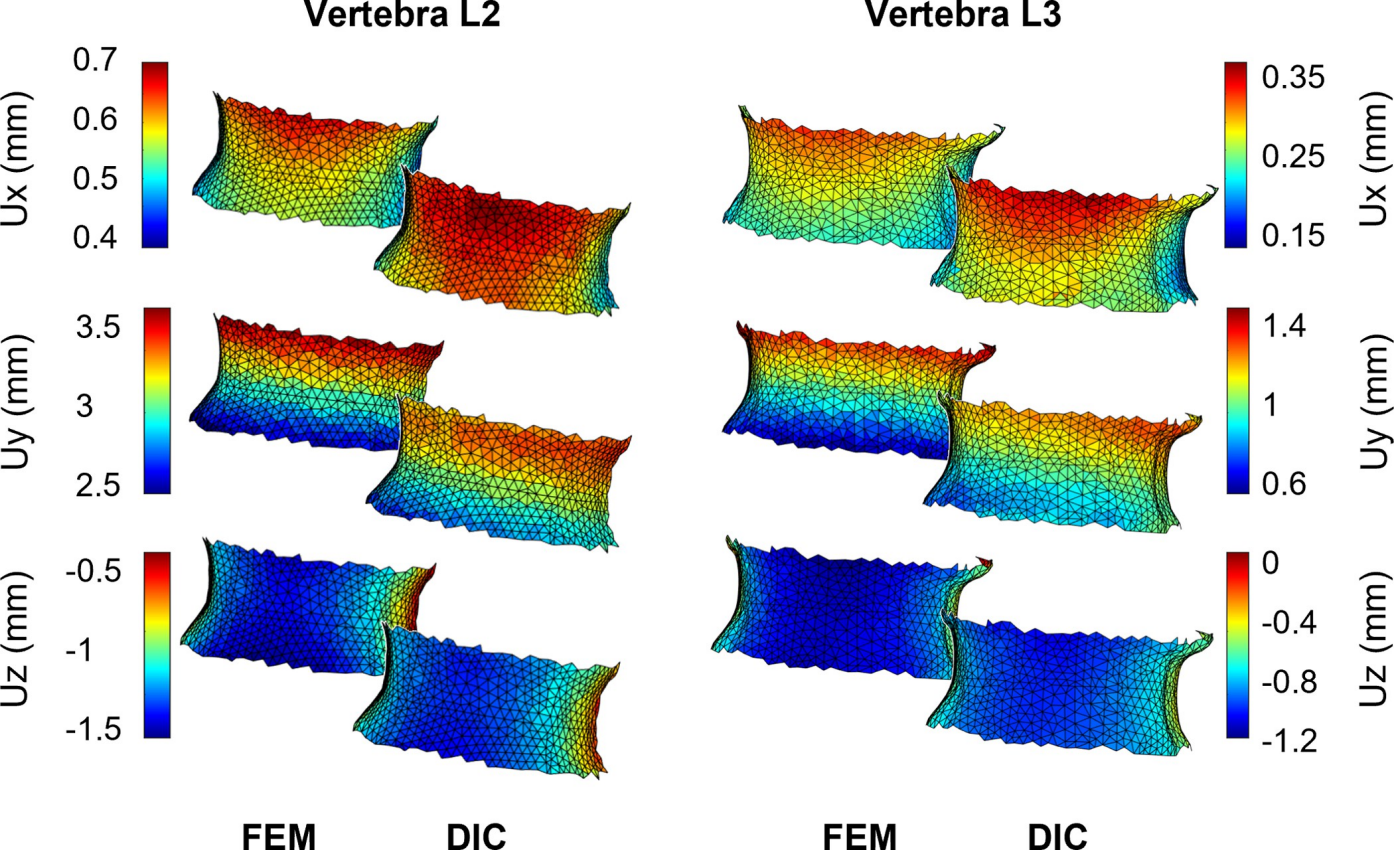
Displacement field of the two vertebral surfaces. Comparison between computational and experimental displacement fields in the three directional components X, Y and Z for each vertebra (L2 left, L3 right). To help the reader in appreciating the comparison, both experimental and computational displacements have been interpolated over the same mesh.

The FE model was able to predict the measured displacements at a load of 60 N with a determination coefficient higher than 0.9 and RMSE less than 0.12 mm (%RMSE less than 7%). A summary of the validation results for the three displacement components are reported in Table 1 while the correlations between the experimentally measured and numerically predicted displacements are shown in Fig 4. Highest average percentage errors were found on the right-left and the superior-inferior direction of the displacement components for the lower vertebra (about 8.7% and 14% respectively). The %RMSE in all the directions was found to be in the range between 1.8% and 7.5%. The magnitude of the error did not seem to depend on the magnitude of the measurements, as can be observed in Fig 5 while the plots of the spatial distribution of the vector resultant (Diff) in Fig 6 highlighted a trend with higher errors on the left side. Finally, spatial distributions of DIC measured displacement fields and FE predicted ones are reported to show the ability of the model to reproduce the realistic spine kinematics (Fig 7).

**Table 1:** Summary of the validation metrics values for local displacement comparisons between DIC and FE results.

	L2			L3		
	RL	AP	SI	RL	AP	SI
R^2^	0.98	0.98	0.98	0.97	0.94	0.98
Slope	0.823	0.976	1.073	0.650	0.979	1.071
Intercept	0.054	-0.030	0.054	0.071	-0.023	-0.038
RMSE (mm)	0.055	0.110	0.065	0.028	0.072	0.105
RMSE %	7.5	2.7	3.7	3.8	1.8	6.0
Average Error %	8.7	3.4	3.9	9.4	6.4	14.0
Maximum Error (mm)	0.077	0.225	0.165	0.079	0.181	0.203

Validation metrics for L2 and L3 vertebral surfaces and calculated on the right-left (RL), antero-posterior (AP) and superior- inferior (SI) displacement components.

## Discussion

The aim of this paper was to develop a first model validation framework based on the comparison between the vertebral surface displacements predicted by the FE model and the experimentally measured ones using DIC for the same spine segment which included an osteoporotic vertebra and a vertebra with lytic metastatic lesions.

To achieve this goal, the first challenge was to align the experimental DIC data to the geometry of the FE model. The proposed registration method allowed to obtained good registration results, comparable with those obtained in the state-of-the-art validation studies [15, 39]. As already mentioned, the radius *R* of the spherical volume of interest defined to compute the average of the measured displacements was set equal to the registration RMSE. In a preliminary sensitivity analysis, it has been noticed that this distance ensured that more than 80% of DIC points had at least a corresponding node of the FE model to be compared with. Also, points whose distance from the closest FE node is more than 0.53 mm were discarded minimizing the influence of the registration errors on the validation results.

The local comparison between the displacements predicted by the developed FE model and the ones measured by the DIC showed robust correlation. The *R*^*2*^ values along the three directions ranged from 0.94 to 0.98 and RMSE % was found always less than 8%. Highest RMSE % value (7.5%) was observed in the RoI of the lesioned vertebra and related to the right-left displacement direction; nonetheless maximum displacement errors and DIC uncertainty for this displacement component were found of the same magnitude order. The systematic procedure developed to define the boundary conditions proved to be overall efficient to represent the experimental loading conditions and obtain good displacement prediction accuracy.

To the author’s best knowledge, no other studies focused on the experimental validation of FE models of human spine segment that include more than two spinal units using DIC. The only study that applied a similar validation approach to FE models of single thoracolumbar vertebral bodies was presented by Gustafson et al. [15]. In their work, a registration method based on the additional acquisition of control points coordinates using a 3D point digitizer was used and the maximum distance between the DIC surface points and FE nodes after the registration was found about 2 mm. The *R*^*2*^ correlation values between the DIC and FE displacements ranged from 0.75 to 0.93. Nevertheless, it is important to notice that in their study individual vertebrae were tested and the overall spine motion, with the role of the IVD in particular, could not be considered in the displacement comparison. Other studies used DIC data to validate FE models of bones (e.g., cadaveric femora and pelvis) showing good predictive accuracy in term of both displacement and strain measurement comparison [22, 23, 40]. Commonly used registration methods relied on landmark-based alignment techniques and iterative closest point algorithm; however, these techniques cannot be easily applied to the antero-lateral surface of the vertebral body as it does not have clearly distinguishing features.

The attempt to replicate *in silico* what happen during experimental tests obviously brings its own set of limitations. A first critical point concerns the mechanical properties assigned to the IVDs. The effect of considering more complex modelling (e.g., nonlinear material properties of the nuclear pulposus and annulus fibrosus) on the simulation outputs is worth studying in future work, that will include a strain-based validation in the workflow. A systematic analysis on the biomechanical response of the discs at different degenerative stages might be required as well as a detailed reconstruction of the IVDs geometry that cannot be easily performed from clinical CT images of the specimen. More complex finite element models will be also needed to investigate the effect of the posterior ligaments and the facet joints that were not included in this work.

Regarding the BCs, one assumption made was that the metal and the PMMA pots were considered as a single rigid body connected to the surface of the vertebral body. This is a realistic assumption because the stiffness of the metal pots is much greater than that of the specimen, and screws ensured a stable connection of the parts. Another important aspect that should be mentioned is that the applied rotations and translations were defined in the coordinate reference system of the FE model considering the transformation matrix extracted after the registration step. This might have introduced some errors due to possible relative motions between adjacent vertebral bodies caused by the insertion of the specimen in the testing machine.

Material properties of the bone were mapped using the same constitutive laws both for osteoporotic and lesioned vertebrae. This choice could have introduced some inaccuracy but was supported by other authors findings [34, 35], who had shown that the mechanical properties for trabecular bone with and without lesions are similar. However, it should be mentioned that even though not clear blastic formations were identified, the lytic regions were widely spread within the L2 vertebral body and very small and higher density regions were found in other parts of the bone segment that might be also caused by an early onset of fracture. In general, the proposed validation method should be considered valid as long as the hypothesis that the metastatic lesions can be characterized as low-density bone tissue remains true. If evident signs of blastic metastases or primary tumours (e.g., osteosarcoma) are observed, other constitutive models should be included to characterize the mechanical properties of the bone tissue.

Last limitation that is important to underline is that the present work has been developed using one single specimen. Nevertheless, this is a first proof-of-concept validation study that allowed to highlight strengths and weaknesses of the methodology. Despite the limitations, it represents a fundamental step towards the application of the technique to more complex models that can be used to predict bone surface strain and assess fracture risk in pathological vertebrae.

In conclusion, a subject-specific finite element model of a lumbar spine segment with mixed metastasis was developed and validated against experimental displacements measured through digital image correlation technique. With the proposed methodology good predictivity accuracy (RMSE% < 8% and R^2^ > 0.9) was achieved in the simulation of the spine kinematics. The present findings show how the experimental spine displacement field could be adequately reproduced by the developed multi-vertebrae FE model, despite the simplified mechanical modelling of the IVDs.
